# Revisiting genital leptospirosis in large animals: impacts on reproductive health, diagnostic challenges, and future directions

**DOI:** 10.1128/jcm.00525-25

**Published:** 2025-12-04

**Authors:** Ana Luiza dos Santos Baptista Borges, Luiza Aymée, Walter Lilenbaum, Maria Isabel Nogueira Di Azevedo

**Affiliations:** 1 Laboratory of Veterinary Bacteriology, Biomedical Institute, Federal Fluminense University 28110, Niterói, Rio de Janeiro, Brazil; 2 Laboratory of Investigation in Medical Microbiology, Institute of Microbiology, Federal University of Rio de Janeiro 28125, Rio de Janeiro, Brazil; Vanderbilt University Medical Center, Nashville, Tennessee, USA

**Keywords:** leptospirosis, diagnostic tools, reproduction, uterus

## Abstract

Leptospirosis, caused by *Leptospira* spp. infection, is a globally significant zoonotic disease that affects a wide range of animals. Although renal colonization is well-documented, genital infection by leptospires remained less explored for decades, despite its impact on reproduction. Evidence suggests that genital infection occurs as a primary condition rather than secondary to renal colonization, particularly in cattle suffering from bovine genital leptospirosis (BGL), linked to chronic infections by strains of the Sejroe serogroup. In horses, a similar condition is suggested to be associated with strains of serogroup Australis. Molecular studies confirmed the presence of *Leptospira* DNA in uterine, follicular, and vaginal samples, strengthening the hypothesis of an independent genital physiopathology. Despite significant advances in molecular diagnostics, the detection of genital carriers remains challenging, requiring refined methodologies beyond standard serology. This review critically examines the historical detection of *Leptospira* spp. in genital samples of cattle, small ruminants, swine, and equines, emphasizing its relevance to reproductive health. Moreover, we highlight the limitations of current diagnostic approaches, advocating for increased use of genital samples in leptospirosis research of large animals and shedding light on future directions regarding genital leptospirosis in livestock research. Enhanced understanding and diagnosis of genital leptospirosis will contribute to better livestock reproductive management and disease prevention.

## INTRODUCTION

Leptospirosis is a bacterial disease caused by pathogenic species of the genus *Leptospira*, affecting a wide range of hosts, including humans (1). This zoonotic disease holds significant relevance within the One Health framework, particularly in tropical and subtropical regions where it is endemic (2). The genus *Leptospira* belongs to the order Spirochaetales, family Leptospiraceae (3), which are recognized as slow-growing, microaerophilic bacteria (4). This group has a spiral-shaped, long, and thin morphology. The periplasmic flagella exhibit a unique structure responsible for the characteristic high motility of *Leptospira*, enabling both translational and nontranslational movements (5). Although leptospires possess a double-membrane cell wall characteristic of gram-negative bacteria, they are poorly visualized by Gram staining due to their thin structure (6). Their outer membrane layer consists of phospholipids, various lipoproteins, and lipopolysaccharides (LPS), which play crucial roles in bacterial pathogenesis and host-bacterial interactions.

To date, the classification of the genus *Leptospira* is based on two distinct but complementary systems: serological and genetic (7). The serological classification is based on the antigenic properties of lipopolysaccharides (LPS). In this classification, *Leptospira* are grouped into serovars, which are further organized into serogroups (8). Historically, the genus was divided into two species based on the specific phenotypic characteristics: *L. interrogans* (pathogenic strains) and *L. biflexa* (saprophytic strains) (3). In contrast, the current genetic classification relies on molecular taxonomy for species identification. It is structured into subgroups designated as P1 (*n* = 17), P2 (*n* = 21), S1 (*n* = 21), and S2 (*n* = 5), which broadly correspond to the formerly defined pathogenic, intermediate, and saprophytic groups (9, 10). These two classification systems are not entirely congruent; therefore, their integrative application is recommended for diagnostic and epidemiological purposes.

The genus *Leptospira* has a worldwide distribution (5). *Leptospira* strains are classified according to the infected host, generally categorized as host-adapted or incidental (11). Serovars Pomona and Grippotyphosa are incidental in horses, while Bratislava is host-adapted (12). Pomona is host-adapted to swine, whereas Icterohaemorrhagiae is commonly incidental (13). In small ruminants, such as sheep and goats, Hardjo (strains Hardjoprajitno and Hardjobovis) may act as adapted strains, while Pomona and Icterohaemorrhagiae are generally incidental (14). Finally, Hardjo is well recognized as host-adapted to cattle, while Icterohaemorrhagiae and Grippotyphosa often act as incidental agents (15).

The clinical manifestation of leptospirosis in animals is influenced by the infecting serovar and interaction with the host species (16). Infections by incidental serovars often lead to acute manifestations, characterized by signs of multiple organ failure (17). Those infections are common in humans and dogs, and occasionally in horses and pigs, but are less frequent in cattle. In the case of large animals, incidental strains lead to abortion, appearing as outbreaks. Conversely, infections caused by host-adapted serovars are typically silent in adult animals. Those infections are mainly associated with chronic reproductive disorders, such as subfertility (Table 1) (18). A syndrome characterized by genital infection with host-adapted leptospires leading to reproductive signs independent of renal colonization has been formally described in cattle and designated as bovine genital leptospirosis (BGL) (19). Consequently, the reproductive syndrome is better characterized in cattle; nevertheless, despite the scarcity of studies, it is presumed that analogous manifestations may also affect other species, including horses (20) and pigs (21).

**TABLE 1:** Discrimination of genital leptospirosis in livestock by main leptospiral agents and reproductive impacts across animal species

Animal species	Leptospires (species and serovars/serogroups)	Reproductive impact	References
Ruminants (bovine, ovine, and caprine)	*Leptospira interrogans* serovar Hardjoprajitno; *L. borgpetersenii* serovar Hardjobovis; *L. santarosai* serovar Guaricura	Embryonic mortality, estrus repetition, subfertility, and infertility. Abortion, stillbirth, birth of weak animals, and fetal mummification.	(11, 14, 15, 18, 19, 22–48)
Equine	*Leptospira interrogans* serovar Bratislava	Embryonic mortality, estrus repetition, placentitis, abortion, stillbirth, and birth of weak foals.	(12, 18, 20, 49–52)
Swine	*Leptospira interrogans* serogroup Australis; serogroup Pomona	Embryonic mortality, estrus repetition, placentitis, abortion, stillbirth, birth of weak piglets, and reduced litter size.	(13, 18, 21, 53–60)

In ruminants, the main agents associated with genital infection are *L. interrogans* (sv Hardjo, strain Hardjoprajitno) and *L. borgpetersenii interrogans* (sv Hardjo, strain Hardjobovis), belonging to serogroup Sejroe, but infections by *L. santarosai* (sv Guaricura) are also important. Strains of *L. santarosai* sv Guaricura are considered emergent in the Americas, not only in cattle (22) but also in humans (61). Recently, a molecular epidemiology study of leptospiral strains linked to adapted serovars from cattle has shown different haplogroups depending on the collection site (renal vs genital) (23). Besides that, Di Azevedo et al. (24) recently demonstrated, based on comparative genomic analyzes, that genital strains of *L. santarosai* express a higher number of virulence factors compared to urinary strains, which reinforces the evolutionary adaptation of some strains to reproductive sites.

Equine genital leptospirosis (EGL) was first introduced in 2022 as a chronic and subclinical disease affecting the genital tract of horses, similar to BGL. The primary infectious agent is most probably *Leptospira interrogans* serogroup Australis, serovar Bratislava, specifically adapted to horses (20). Unlike the diseases caused by incidental strains that lead to acute symptoms in horses, as the systemic, ocular, and pulmonary syndromes, the host-adapted strains of sv Bratislava typically result in subclinical disease confined to the genital tract. Because the signs are not readily apparent, such as estrus repetition and reduced fertility, the disease is frequently overlooked and underdiagnosed.

Leptospirosis significantly affects livestock production due to its reproductive impact (Table 1), leading to substantial economic losses (25). Although quantitative estimates are scarce, recurrent reproductive failures, reduced conception rates, and increased culling and insemination costs have been consistently reported in bovine leptospirosis (22, 25, 26), indicating significant productivity losses comparable to those observed in other bacterial reproductive diseases such as brucellosis (62). In this context, accurate diagnosis, prevention, and control measures become essential to reducing the incidence of the disease and mitigating its reproductive impacts. The intricate epidemiological dynamics of leptospirosis make disease control particularly challenging (63). Currently, control programs advocate an integrated “triad” of interventions: antibiotic therapy, whole-cell bacterin vaccination, and environmental management, including quarantine of newly introduced animals and isolation of identified shedders (64). Yet large-scale antibiotic use elevates production costs and faces restrictions on its use in key markets such as the United States and the European Union due to concerns over antimicrobial resistance and drug residues in meat and milk (65).

Vaccination remains the most cost-effective preventive strategy (66), but current commercial bacterins rely on international renal reference strains whose antigenic composition may not reflect local serogroup diversity (22, 67). This mismatch can limit protective efficacy, underscoring the imperative to obtain and characterize regional *Leptospira* spp. populations. The molecular and serological characterization of locally circulating strains may support selecting and developing area-specific vaccines with maximal field performance.

Currently, the most common method of diagnosis of leptospirosis relies on serology, mainly the Microscopic Agglutination Test (MAT) (68). However, since chronically infected animals may present with low titers or even seronegative results, molecular diagnostic tools are more effective in identifying the carriers. Given the intricate physiopathology of the leptospiral infection, it is essential to determine the specific location of the bacteria within the host at any given moment. It allows a thorough understanding of the physiopathology of genital infection, which is essential for selecting the appropriate sample and establishing an accurate diagnosis as the optimal sample depends on the stage of the disease.

Despite its implications for reproductive performance, economic impact, and disease transmission, genital leptospirosis remains a neglected topic in animal health research. Researches worldwide still prioritize renal sites in leptospirosis in livestock, even though the first genital strain was recovered from a cow in 1986 (27). This can be explained by the fact that leptospirosis is traditionally associated with renal shedding, and sampling of genital sites in live animals, particularly uterine fragments, may be challenging. Despite these difficulties, emerging evidence indicates that genital colonization by leptospires can occur independently of renal shedding. This finding underscores the importance of considering genital tract infections even when renal samples or urine are negative. Among the available methods for investigating genital leptospirosis, the collection of cervicovaginal mucus (CVM) stands out as a practical and effective choice (28).

In this context, the present review critically evaluates and synthesizes current knowledge on the colonization and identification of *Leptospira* spp. in the genital tract of large animals, including cattle, small ruminants, swine, and equines, emphasizing its relevance to reproductive health. Rather than presenting new experimental data, this review consolidates fragmented and historically overlooked evidence, integrating findings across species and anatomical sites to provide a comprehensive framework that identifies key research gaps and outlines future directions for diagnostic refinement, epidemiological surveillance, and reproductive health management in livestock.

## METHODOLOGICAL APPROACH

This review was based on an extensive literature search conducted between March 2024 and July 2025 across the PubMed, Scopus, and Web of Science databases, using combinations of the keywords “Leptospira,” “genital leptospirosis,” “bovine,” “equine,” “swine,” and “small ruminants.” Inclusion criteria comprised peer-reviewed studies reporting detection, isolation, or experimental infection of *Leptospira* spp. in genital samples of large animals. Gray literature and studies lacking methodological detail were excluded. Although narrative in nature, this review followed structured selection criteria to enhance the transparency and reproducibility. Based on this, this review was structured around three analytical dimensions: (i) the physiopathological mechanisms distinguishing genital from renal colonization; (ii) the comparative patterns of infection across major livestock species; (iii) the diagnostic and epidemiological implications of genital infection within a One Health context.

## PHYSIOPATHOLOGY OF LEPTOSPIROSIS

The bacterium enters through injured or intact mucosa, using its highly motile endoflagellum proteins to invade the host (69, 70). Transmission occurs either by direct contact or via contaminated environments, allowing the pathogen to reach the bloodstream, a phase called leptospiremia (3). Once the immune response clears much of the circulating bacteria, the infection enters the second phase, known as leptospiruria. In this second phase, *Leptospira* spp. persist in not only the renal tubules but also in other immunoprivileged organs, such as the genital tract (71). It is well-known that renal colonization plays a crucial role in bacterial shedding in urine, making those animals a potential source of infection and environmental contamination (72). In this case, leptospires tend to colonize the proximal convoluted tubules, leading to a chronic kidney infection (5). In large animals, it is known that host-adapted strains can also colonize the genital tract and cause chronic infection, resulting in subclinical signs mainly in the reproductive sphere (11).

The exact physiopathology of genital leptospirosis is not yet well understood. For many years, it was believed that genital colonization would occur as a minor and secondary effect to the renal infection; however, there is evidence that the genital tract is an important primary site of infection itself, even when the kidneys are not colonized (19). The confirmation of the bacterium in genital sites, such as CVM, uterus, follicles, ovaries, and placenta, reinforces the importance of leptospiral colonization in the genital tract (29–31). In addition, there is evidence that colonization of *Leptospira* spp. in the genital tract is associated with a local immune response since specific antibodies have been identified in CVM samples from naturally infected cows (73). Despite the increasing evidence for primary genital colonization, some studies still suggest a secondary role following renal infection (72, 74). These conflicting interpretations highlight the need for longitudinal and experimental studies to clarify the dynamics of leptospiral tissue tropism.

Some of the hypotheses that try to explain the reproductive losses, such as estrus repetition, embryonic reabsorption, infertility, and birth of weak animals, include both the direct action of the bacterium on the embryos and the indirect action, through the modulation of the uterine environment, making it inhospitable to embryo implantation (19). Direct action could occur through the penetration of the bacterium, causing damage to the embryos and preventing their complete formation. Confirmation of the presence of leptospires in the follicular fluid of naturally infected animals supports the hypothesis that the infection occurs even before fertilization and may compromise embryonic formation (31). On the other hand, the indirect action occurs through inflammation in the uterine environment, which would impair embryo implantation. Inflammatory cytokines (such as IL-6) were detected in uterine samples from naturally infected cows, reinforcing that hypothesis (32). Therefore, evidence suggests that both direct and indirect actions can contribute to the reproductive losses related to genital leptospirosis.

## A HISTORICAL OVERVIEW OF *LEPTOSPIRA* SPP. IN ANIMAL GENITAL SAMPLES

The first genital strain of *Leptospira* spp. was isolated from a cow in 1986 (27). Nevertheless, scientific research focusing on the genital tract of livestock has only expanded significantly in the past decade. While much of the recent progress in understanding genital leptospirosis has originated from South American research groups, particularly Brazil, where large-scale cattle and pig herds, tropical and subtropical environmental conditions, and well-established veterinary research networks have facilitated investigations, similar, though fewer, pieces of evidence are found elsewhere, including North America, Europe, and Asia. For example, surveillance of swine in Germany reported that *Leptospira* can persist in both the kidneys and genital tract and be excreted in genital fluids (53). A European systematic review of bovine leptospirosis acknowledged that the genital tract represents a major extrarenal colonization site (11). Historical and regional data thus underscore that genital colonization by *Leptospira* is not regionally restricted. Under-recognition outside Latin America may reflect research emphasis and field conditions rather than the true absence of the phenomenon, which is encouraged to be further investigated. This broadens the view of genital leptospirosis as a widespread, underdiagnosed syndrome with significant veterinary implications worldwide.

The next subsections present the main findings regarding leptospiral strains identified in livestock species: ruminants, equines, and swine. It is important to note that the amount of available information varies markedly among species. Most published studies on genital leptospirosis concern cattle, where the syndrome has been formally recognized and investigated under both field and experimental conditions. In contrast, reports on horses, swine, and small ruminants remain scarce, often limited to isolated case studies or small sample sets. This imbalance reflects gaps in surveillance rather than true biological differences and underscores the need for targeted research in underrepresented species.

### Cattle

Despite the explicit clinical signs related to reproductive failures, most studies have focused on the detection of leptospires or their DNA in urine (16, 75, 76), while studies on the infective strains of *Leptospira* spp. in the reproductive tract of cows have received less attention (22). A recent study has shown that urine is not an adequate sample for diagnosing leptospirosis in animals experiencing reproductive losses. In this research, 106 nonpregnant cows with poor reproductive performance were examined, and 73 (68.9%) were found to be infected. However, only 14 of the infections (19.2%) were diagnosed using exclusively urine samples. In contrast, 64 infections (87.7%) were diagnosed through genital samples, which underscores the significance of this colonization site (28).

The bacterium was first identified on the genital tract of nonpregnant cows about 40 years ago (27) and confirmed in a larger study, when the authors reported that the genital tract appeared to be an important extrarenal site (77). In these studies, the strains were identified as *L. interrogans* serovar Hardjo and were isolated from the uterus and oviduct in samples collected in a slaughterhouse. Subsequently, in a study involving vaccination and challenge of cows with the adapted strain *L. interrogans* serovar Hardjo, leptospires were identified in the uterus and oviduct of both vaccinated and unvaccinated cows (78). Using indirect immunofluorescence, Dhaliwal and colleagues (73, 79) identified leptospires in CVMs of naturally infected cows, as well as in heifers experimentally infected with *L. interrogans* serovar Hardjo. With the advent of molecular techniques for the identification of *Leptospira* species, it was possible to identify the presence of DNA in embryos and oocytes of heifers experimentally infected with the strain *L. borgpetersenii* serovar Hardjobovis, and infection by *Leptospira* sp. was also identified in the reproductive tract of these heifers by direct microscopy (33). Despite these important studies, uterine infection has received limited attention, and molecular identification of *Leptospira* spp. in genital tissues remains underexplored. It was only in 2017 that molecular investigations of genital infection in cattle were resumed (34), when a large-scale study detected *Leptospira* DNA in 50.4% of vaginal fluid samples collected from slaughtered cows. Additionally, using paraffin-embedded bovine uterine tissues stored for 13 years, Pires et al. (80) reported PCR positivity in 18% of samples.

Based on the increasing evidence of the important genital colonization by leptospires, Loureiro and Lilenbaum (19) published a comprehensive review addressing key aspects of this syndrome, including methodologies for genital sample collection. The publication describes the technique of collecting CVM in the vaginal fornix region, using a brush attached to a cytology device, avoiding contact with the urinary meatus, and preventing possible contamination of the sample by urine.

After the publication of the BGL review, several studies have identified and molecularly characterized *L. interrogans* serogroup Sejroe in the uterus of slaughtered cows (35); follicular fluid of non-pregnant cows (36, 37); linked reproductive disorders in live subfertile cows under field conditions to infection by *L. interrogans*, *L. noguchii*, *L. santarosai*, and *L. borgpetersenii* (38, 39); and demonstrated the role of *L. santarosai* serovar Guaricura as a causative agent of BGL (40).

Other research groups have also identified *Leptospira* sp. in clinical samples of vaginal mucus in naturally infected cows in the field (29), in addition to vaginal fluid, uterus, placenta, and fallopian tube from cows slaughtered in Northern Brazil (41). Still, in the same region, the same group demonstrated the transplacental infection of *Leptospira* sp. in samples of fetuses and cows (30). Positive genital samples in PCR were vaginal fluid, uterus, placenta, fallopian tube, and ovary. In fetuses, samples were positive in the central nervous system, lung, peritoneal fluid, abomasal content, liver, spleen, bladder, kidney, and reproductive tract.

Culturing genital pathogenic strains is still a challenge. In addition to those recovered from the genital tract of cows in 1986, they were isolated from CVM samples from cows and sheep (42, 43) and from the uterus (40). The cow uterine isolate was characterized as *L. santarosai* serovar Guaricura, while two strains recovered from bovine CVM were characterized as *L. santarosai* serogroup Sejroe (44) and one as *L. noguchii*. In addition, Loureiro et al. (34) also identified strains belonging to *L. meyeri* and *L. alstonii* obtained from vaginal fluid of cows in slaughterhouses. Other research groups have identified *Leptospira* sp. in cultures of genital samples, including uterine fragments, placenta, vaginal fluid, and ovary (30). In this study, the ovarian strain was characterized as *L. borgpetersenii*. Recently, an isolate was recovered from the follicular fluid of a naturally infected cow, which was characterized as *L. interrogans* sg. Icterohaemorrhagiae, a highly virulent strain with zoonotic potential (81).

Regarding genital male samples, the presence of *Leptospira* spp. has been detected by molecular tools in semen, testicles, and epididymis (82, 83). Although bulls play a critical role in the venereal transmission of leptospirosis, they remain understudied, and their contribution to genital leptospirosis remains to be understood. Consequently, future research should focus on infections of the male genital tract, recognizing that a single infected bull may transmit the disease to an entire herd. Such investigations are essential for developing targeted reproductive management measures to reduce infection.

### Small ruminants

Due to their small size and lower maintenance cost compared to cattle, small ruminants, especially sheep, have been used as experimental biomodels for bovine reproductive syndrome. Besides being used as biomodels in experimental research, small ruminants are also naturally infected by leptospires in genital sites. Similar to cows, the reproductive issues in sheep include abortions, fetal mummification, stillbirth, and birth of weak animals (45). Leptospirosis is less common in goats, which are more resistant than other species, though reproductive problems have been noted (46). The exposure to wild animals or co-grazing with other species may increase the risk of leptospirosis in small ruminants (84). The disease reduces productivity due to reproductive losses, impacting livestock economics (47).

Regarding naturally infected animals, the recovery of pathogenic species of *Leptospira* from vaginal fluid samples from sheep and goats was reported (85). More than 10 years later, another study described the genital infection of sheep, finding a positivity of 37.5% on slaughtered animals (86). In 2022, Santos and collaborators (48) evaluated vaginal fluid samples from sheep raised in semi-intensive production systems in the Brazilian semi-arid region, and 83.3% were PCR-positive, and 13.3% in the bacteriological culture. The sequencing of two samples obtained from vaginal fluid revealed *L. santarosai* and *L. interrogans*.

Subsequently, the efficacy of streptomycin therapy for genital leptospiral infection was evaluated in experimentally infected sheep (87) and later reproduced in naturally infected cows (88). These studies demonstrated a reduction in the genital carrier status following a treatment protocol of 25 mg/kg administered for 3 consecutive days. However, comparable investigations using alternative antimicrobials have not yet been conducted. Therefore, although these findings are promising, they cannot yet be generalized to other nonruminant hosts or extrapolated to different geographical regions, particularly because streptomycin is not approved for use worldwide.

The impact of vaccination in preventing genital colonization in experimentally infected sheep was also evaluated (89). Although renal colonization disappeared after 120 days, genital colonization persisted for at least 210 days post-infection. These findings underscore the challenges inherent to controlling genital leptospirosis in ruminants. Although significant advances have been made in understanding the disease, prevention strategies have largely been evaluated in isolation. Therefore, there is a critical need for integrated prophylactic approaches that enable a more comprehensive and effective control of genital leptospirosis in these animals.

Recently, an important study aimed to evaluate the efficacy of the genital route in inducing *Leptospira* spp. infection in sheep (90). For this, monthly samples of cervicovaginal mucus and uterine fragments were collected for molecular detection by PCR. The results showed that all animals were effectively infected, reinforcing the genital transmission as a natural and efficient route of infection.

### Swine

The identification of *Leptospira* spp. in the genital tract of pigs was first described in the late 1980 s, similarly to cattle, with the isolation of *L. interrogans* serovar Bratislava from the genital tract of slaughtered sows (54). In the same year, the same group also isolated strains of serogroup Australis and serovars Muenchen and Bratislava from samples of the upper genital tract of sows (55). In 1992, *L. interrogans* serovars Bratislava and Hardjo were isolated from the uterus and oviduct of slaughtered animals (56). More than a decade later, another report of *L. interrogans* from genital samples of slaughtered pigs was characterized as Pomona (57).

More recent studies have focused on the identification of *Leptospira* spp. using molecular techniques such as PCR and qPCR, along with genetic typing. In feral pigs, *Leptospira* spp. were detected by qPCR in samples from the testes, epididymis, uterus, placenta, and aborted fetuses. Furthermore, isolation of *L. interrogans* serogroup Australis and *L. kirschneri* serogroup Grippotyphosa was reported from male genital samples (91). Another study detected pathogenic leptospires in vaginal fluid using PCR (92), and, most recently, CVM samples from nonpregnant sows were analyzed by PCR and strains genetically characterized as *L. interrogans*, similar to strains of serogroup Australis (21).

Seroreactivity to leptospirosis has been associated with reproductive losses in swine (58). Despite the economic impact caused by the disease, the investigations regarding leptospirosis in swine are still scarce (21). The main clinical signs are the occurrence of abortions and stillbirths and a reduction in the number of piglets (59). The presence of leptospires in the genital tract suggests that venereal transmission is an important route of infection in these animals (60). Further research is needed to identify the primary strains in the genital tract and to develop better control measures and elucidate the impacts of the presence of *Leptospira* spp. in the swine genital tract.

### Equine

The identification of pathogenic leptospires in genital samples of mares has received greater attention recently, mainly based on molecular direct detection methods. In 2014, a study detected *Leptospira* DNA in vaginal fluid samples from mares, and sequencing revealed *L. interrogans* closely related to strains of the Australis serogroup. The following year, the same group confirmed its presence in uterine and vaginal fluid samples using qPCR, complemented by indirect immunofluorescence assays. Sequencing revealed *L. interrogans* (Bratislava and Pomona) and *L. borgpetersenii* (Hardjobovis) (49, 50).

With increasing evidence of leptospirosis affecting the genital tract of horses, Di Azevedo and Lilenbaum (20) published a review addressing the main factors involved in the reproductive syndrome caused by *Leptospira* spp. Although research on genital infection in cattle has produced substantially more data, it is plausible that the reproductive syndrome in horses, so-called EGL, occurs similarly to that observed in cattle. Supporting this, in 2024, *L. interrogans* was identified in cervicovaginal mucus (CVM) samples by PCR (51), and in 2025, by qPCR in endometrial swab samples (52).

EGL is a neglected, silent, and chronic disease characterized by reproductive issues such as subfertility, placentitis, abortions, or the birth of weak foals (51). Recognizing the genital site as a significant colonization area is crucial for implementing management strategies that can mitigate reproductive losses and, consequently, reduce the economic impact of the disease. Therefore, EGL requires greater attention, and leptospirosis diagnosis should be included in the reproductive management protocols for mares, mainly based on the PCR of genital samples such as CVM.

## DIAGNOSTIC APPROACHES FOR GENITAL LEPTOSPIROSIS: CURRENT METHODS AND LIMITATIONS

The absence of evident symptoms of the silent and reproductive form of genital leptospirosis consists of a great challenge for diagnosis, due to its chronic characteristic, which is commonly underdiagnosed (93). Genital leptospirosis is a little-known disease and has been largely neglected by researchers; therefore, there is a critical need for the development of more efficient diagnostic methods. Information about diagnostic methodologies and sampling collection for genital leptospirosis investigations is provided in Table 2.

**TABLE 2:** Diagnostic tools for genital leptospirosis

Diagnostic tool	Sample	Specificity	Sensitivity	Advantages	Limitation
Culturing	Urine, uterus, and CVM	High	Low	Epidemiological purposes and vaccine composition	Fastidious growth, nutritional requirements, and high cost
MAT	Blood	Variable	Variable^*a*^	Herd screening	Failure to identify individual carriers, cross-reaction with previous infection and vaccination antibodies, and requires specialized training and live cultures
PCR	Urine	High	Variable	Identify carriers and differentiate species	Intermittent urinary shedding
	Uterus	High	High	Identify carriers and differentiate species and site of infection	Sample collection
	CVM	High	High	Identify carriers and differentiate species and more practical sample collection	Possible lower bacterial load when compared to uterus

^*a*^ Can be improved by using local strains in the antigen panel.

Direct detection of leptospires in fresh samples is not commonly used. Besides being quite subjective and with low sensitivity, due to the thickness of its cell wall, the bacterium is not stained by the Gram stain, requiring darkfield or phase-contrast microscopy for its visualization (94). Besides, the minimum concentration of bacteria in the sample to allow the visualization in dark-field microscopy is 10^4^ leptospires/mL (95). Another form of bacterial direct identification is culturing. Nevertheless, due to the fastidious growth and nutritional requirements, its use for diagnosis is limited (96). However, it has epidemiological importance since it is the only method capable of confirming the infecting strain of the host. Besides, isolates can be incorporated into the antigen panel for MAT, increasing sensitivity and being used for the composition of vaccines (97, 98).

Currently, the most common method of diagnosis of leptospirosis is based on serology, mainly MAT, and direct methods, such as PCR (68). The applicability of MAT is restricted to epidemiological purposes, especially for herd screening, but not for individual diagnosis (68). Recent studies have indicated that MAT alone cannot identify individual genital carriers, especially in cases of infection by adapted strains with low titers (29, 99). Recently, it was demonstrated that, even adopting a cut-off point as low as 100 on the MAT, the specificity and sensitivity remained low, below 70% (99). Besides, it requires live leptospires cultures to compose the antigenic panel, which are slow growing and highly sensitive to contaminants (100). Specialized training is essential to perform the test due to the inherent subjectivity of the evaluation; consequently, it is primarily performed in specialized laboratories. Another limitation is the standardization of cut-off points, which can vary by region and host species due to differences in endemicity and levels of bacterial exposure.

Molecular methods such as PCR are more efficient for diagnosing chronic silent leptospirosis (101). The main advantages include greater sensitivity and specificity, not requiring live bacteria, and being used for various clinical samples. Currently, the most used target gene for the pathogenic leptospires detection is *lipL32*, found abundantly in the outer wall of these bacteria (68). Other targets may also be used, such as the *flaB* and *rrs* genes (102, 103). One of the main challenges in molecular diagnosis is selecting a target specific to pathogenic *Leptospira* that ensures high amplification sensitivity. Additionally, molecular characterization often falls short in accurately predicting the circulating serogroups and serovars, which are critical for devising effective, region-specific control measures, such as vaccination strategies.

Traditionally, samples of the urinary tract of animals, such as urine, have been used to diagnose leptospirosis in animals with reproductive disorders (16, 76). However, it has been demonstrated that samples from the genital sites, as CVM, are more suitable for diagnosing genital leptospirosis (28). Therefore, genital samples should be prioritized for molecular diagnosis of reproductive disorders caused by leptospirosis.

The diagnostic landscape for genital leptospirosis remains fragmented, with substantial variation in sampling procedures, target genes, and analytical protocols across laboratories. Comparative assessments of diagnostic sensitivity and specificity between methodologies, such as serology, culture, conventional PCR, and qPCR, are still scarce. The absence of harmonized guidelines for sample collection and preservation, particularly for cervicovaginal mucus and uterine tissues, hampers reproducibility among studies. Although culture remains the gold standard for strain identification, the diversity of media formulations and antibiotic cocktails used (e.g., EMJH, Fletcher, or T80/40LH) can lead to inconsistent results. Implementing basic quality control measures, such as inclusion of positive and negative extraction controls, duplicate testing, and cross-validation of molecular assays, should be prioritized to enhance diagnostic reliability.

Considering the current evidence, we strongly recommend testing genital samples, such as cervicovaginal mucus and uterine fragments, for the diagnostic investigation of leptospirosis, particularly in animals with reproductive disorders. Molecular screening targeting the *lipL32* gene can be used as an initial test for the detection of pathogenic *Leptospira* spp., followed by species-level identification using a nested PCR approach based on the *secY* gene, as previously proposed (101). This two-step workflow combines sensitivity and taxonomic resolution, providing a standardized, reproducible strategy that may serve as a reference for both research and diagnostic laboratories. Coordinated efforts to establish and validate such standardized protocols would substantially improve comparability among studies and strengthen epidemiological surveillance worldwide.

## IMPLICATIONS OF USING GENITAL SAMPLES IN THE DIAGNOSIS OF GENITAL LEPTOSPIROSIS

Considering the currently available diagnostic tools, it is suggested that serology and PCR of genital samples, such as uterine fragments or CVM, should be associated for the accurate diagnosis of genital leptospirosis and identification of genital carriers. Investigations have shown that testing samples from the urinary tract, such as urine, is inadequate to efficiently identify genital carriers (28, 34, 41), while CVM and uterine fragments are comparable for identifying the genital carriers (28).

Samples of uterine fragments can provide a superior quality of genetic material, as the uterus is the primary site of infection. However, one limitation of this approach is the difficulty in obtaining samples from live cows, so usually, only small pieces can be collected. Besides, the physiopathology of uterine infection is still poorly understood, and it is unclear if there is a better location in the organ for the collection. Cervicovaginal mucus (CVM), on the other hand, represents a reflection of the uterine environment, and the fluid nature of this biological material facilitates the extraction of microbial DNA from the sample. However, it may still yield false-negative results due to a reduced bacterial load (19). The collection of CVM samples is easier and safer for the animal and is currently widely recommended for diagnosing reproductive problems in cows (19).

## CHALLENGES AND FUTURE PERSPECTIVES

Across the reviewed studies, methodological heterogeneity remains a major challenge. Differences in sample types, molecular targets, and diagnostic thresholds often prevent direct comparison between data sets. Many reports are also limited by small sample sizes or lack quantitative analyses, highlighting the need for standardized experimental designs and multicentric validation. In summary, future research should follow a structured roadmap: (i) short-term: methodological standardization and diagnostic validation; (ii) medium-term: epidemiological expansion, vaccine improvement, and economic evaluation; (iii) long-term: translational integration within One Health frameworks, culminating in policy and regulatory implementation.

### Short-term priorities: standardization of diagnostics and culture protocols

Immediate priorities should focus on methodological harmonization and diagnostic refinement. Comparative analyses of genetic targets for detecting pathogenic *Leptospira* species are still uncommon, hindering the establishment of uniform molecular diagnostic protocols for the genus. Likewise, molecular markers used for species characterization remain poorly standardized, complicating *in silico* analyses and comparative studies across geographic regions. Traditionally, the 16S rRNA (*rrs*) gene was among the first targets employed; however, its low taxonomic resolution limits species differentiation within the genus, reinforcing the need for alternative markers. The *secY* gene currently provides excellent taxonomic resolution (101), while other genes such as *gyrB* and *lfb*-1 can also be applied (104). Although *secY* has lower sensitivity than *lipL32* due to its larger amplicon size, methodological refinements such as nested PCR can improve amplification sensitivity and specificity (101). We strongly recommend the inclusion of genital samples, such as cervicovaginal mucus and uterine fragments, in the diagnostic workflow, particularly for animals with reproductive disorders. A practical molecular strategy combining *lipL32*-based PCR screening followed by *secY*-targeted nested PCR for species identification provides both sensitivity and taxonomic precision. The validation of this workflow through multicentric trials should be a key near-term goal.

Parallel efforts should also target the standardization of culture protocols since the cultivation and identification of *Leptospira* spp. remain poorly harmonized. These bacteria grow slowly, require specific nutritional conditions, and pathogenic strains show limited survival outside animal hosts, which hampers *in vitro* proliferation (105, 106). Multiple culture media, such as EMJH, Fletcher, and T80/40LH, are in use (107), with different antibiotic cocktails to suppress contaminants (108–110). The lack of standardized formulations and subjective assessment of culture growth limits comparability among laboratories. The adoption of molecular confirmation of positive cultures (e.g., PCR-based identification) should be integrated as a quality-control measure to improve accuracy and reproducibility.

### Medium-term priorities: epidemiological, prophylactic, and economic research

Once diagnostic and culture workflows are standardized, the next priority is to expand their application in epidemiological and prophylactic contexts. Epidemiological studies using genital samples are still scarce but are essential in characterizing circulating strains, understanding tissue tropism, and guiding prophylactic measures. Integrating these data into open-access reference databases will facilitate genomic surveillance and cross-regional comparisons.

Concerning disease control, future research should focus on combining vaccination and antibiotic therapy to evaluate their effectiveness in eliminating the carrier status. Current evidence suggests that vaccines do not fully prevent genital colonization and that persistence of *Leptospira* in the genital tract may occur even after vaccination (89). Commercial vaccines, which are based on nongenital strains, may not provide sterilizing immunity at genital sites. Therefore, evaluating vaccines formulated with local genital strains should be prioritized.

Similarly, antimicrobial therapy such as the protocol of 3 days of streptomycin (25 mg/kg) has shown promising results in both experimentally and naturally infected ruminants (87, 88). However, due to the cost, limited feasibility, and unavailability of streptomycin in some countries, alternative antimicrobial agents should also be evaluated (111). Moreover, new treatment protocols should be investigated not only in ruminants but also in swine and equine species.

Formal economic assessments comparing the cost-effectiveness of different diagnostic and control strategies are still lacking. Future studies should quantify the economic impact of incorporating genital sampling into herd surveillance, considering both diagnostic expenses and potential reduction of reproductive losses.

### Long-term priorities: translational and integrative One Health research

In the long term, innovation should focus on translational and integrative research within a One Health framework. Chronic genital carriage contributes to environmental contamination through genital secretions, potentially sustaining infection cycles involving wildlife and peri-domestic reservoirs. The close contact between farmers, veterinarians, and infected livestock also represents an underrecognized occupational hazard in endemic regions.

Integrating reproductive surveillance with environmental and public health systems is therefore essential. Enhanced detection of genital carriers may reduce not only reproductive losses but also the broader zoonotic burden of leptospirosis. Addressing implementation challenges, such as the costs of advanced molecular assays, infrastructure limitations in rural areas, and the need for specialized training, will be crucial for sustainable adoption. Funding mechanisms prioritizing inter-institutional and intersectoral collaborations are necessary to bridge diagnostic research, herd-level management, and zoonotic surveillance.

Translating diagnostic and epidemiological advances into tangible control outcomes will require supportive regulatory and policy frameworks. The diagnostic workflows proposed in this review, particularly the use of *lipL32* screening followed by *secY*-based nested PCR for species identification, should undergo inter-laboratory validation and national accreditation before routine implementation. Harmonization with international guidelines from the World Organization for Animal Health (WOAH) and national veterinary authorities (e.g., United States Department of Agriculture, USDA; European Food Safety Authority, EFSA) will ensure diagnostic traceability, biosafety, and global recognition.

At the policy level, incorporating genital leptospirosis surveillance into existing reproductive health and zoonosis control programs can strengthen early detection and outbreak prevention. Integrating genital sample testing into herd certification and artificial insemination programs may also reduce barriers in animal trade and movement, ensuring compliance with sanitary standards. Implementation success will depend on intersectoral coordination between academic institutions, veterinary services, and public health agencies. The creation of unified case definitions, reporting standards, and data-sharing frameworks will bridge diagnostic innovation with regulatory enforcement and global surveillance efforts.

By aligning these priorities with realistic feasibility, intersectoral collaboration, and supportive regulatory frameworks, genital leptospirosis can transition from a neglected reproductive condition to a recognized model of multidisciplinary zoonotic research and evidence-based policy action.

## CONCLUSION

Genital leptospirosis remains an underestimated yet critical component of the epidemiology of leptospirosis in domestic animals, mainly large animals. The evidence gathered over decades demonstrates that genital colonization by *Leptospira* spp. is not merely occasional but constitutes an independent pathological condition with significant repercussions on reproductive performance, particularly in livestock. Reproductive failures associated with genital leptospirosis can substantially reduce herd productivity, increase replacement costs, and compromise reproductive efficiency, thereby generating significant economic losses at herd and regional levels. Despite advances in molecular diagnostics, the detection of genital carriers is still constrained by methodological limitations and the underutilization of genital samples in routine diagnostics. Addressing these gaps requires the standardization of molecular targets, developing more sensitive and specific diagnostic protocols, improving leptospiral culturing protocols, better vaccination and treatment methods, and finally, a paradigm shift in surveillance strategies to include genital sampling routinely. Integrating genital leptospirosis surveillance into One Health frameworks is essential, given the potential for occupational exposure among veterinarians and farm workers handling infected reproductive tissues. Enhancing our understanding of genital leptospirosis is not only pivotal for improving reproductive management and animal health but also represents a critical step toward more effective disease control and prevention.
